# Flavonoid-Rich Mixed Berries Maintain and Improve Cognitive Function Over a 6 h Period in Young Healthy Adults

**DOI:** 10.3390/nu11112685

**Published:** 2019-11-06

**Authors:** Adrian R. Whyte, Nancy Cheng, Laurie T. Butler, Daniel J. Lamport, Claire M. Williams

**Affiliations:** School of Psychology & Clinical Language Sciences, University of Reading, Earley Gate, Reading, Berkshire, RG6 6AL, UK; a.r.whyte@reading.ac.uk (A.R.W.); nancy.cheng@pgr.reading.ac.uk (N.C.); laurie.butler@anglia.ac.uk (L.T.B.); daniel.lamport@reading.ac.uk (D.J.L.)

**Keywords:** berry, blueberry, raspberry, strawberry, blackberry, polyphenol, flavonoid, cognition, executive function, mood

## Abstract

Research with young adults has previously indicated flavonoid-rich berry interventions facilitate improved executive function (EF) and positive affect 20 min–2 h post-dosing. There has been little consideration of the impact of a berry intervention over a working day and interventions have also tended to consider only a single berry type. This study investigated the temporal profile of EF and mood changes over a 6 h period following a mixed-berry intervention. We hypothesized berry-related benefits would be most evident when participants were cognitively compromised on demanding elements of the task or during periods of fatigue. The study employed a single-blind, randomized, placebo controlled, between-subjects design. Forty participants aged 20–30 years consumed a 400 mL smoothie containing equal blueberry, strawberry, raspberry, and blackberry (*n* = 20) or matched placebo (*n* = 20). Mood was assessed using the Positive and Negative Affect Schedule; EF was tested using the Modified Attention Network (MANT) and Task Switching (TST) Tasks. Testing commenced at baseline then 2, 4 and 6 h post-dosing. As expected, following placebo intervention, performance decreased across the day as participants became cognitively fatigued. However, following berry intervention, participants maintained accuracy on both cognitive tasks up to and including 6 h, and demonstrated quicker response times on the MANT at 2 and 4 h, and TST at 6 h. This study demonstrates the efficacy of flavonoid rich berries in maintaining or improving cognitive performance across the 6 h day.

## 1. Introduction

Diet is an important lifestyle factor having a considerable impact on cognitive development and healthy brain function across the lifespan. One particular dietary component, the flavonoids, have received growing interest for their effects on cognition. Flavonoids are a class of polyphenols found in abundance in a number of foods such as berries, tea, cocoa, citrus fruit, and green leafy spices. Epidemiological studies have indicated that a flavonoid-rich diet is protective against cognitive decline [1,2] and the onset of dementia [3]. Furthermore, there is a growing body of research which demonstrates improved cognitive performance across a number of age groups following both chronic [4] and acute [5] flavonoid-rich interventions.

Berries are a particularly rich source of a flavonoid subclass called anthocyanidins [6,7] and have accordingly been used as a flavonoid-rich intervention in a number of pre-clinical and clinical trials. Pre-clinical trials with both young and aging rodents have found improved cognitive performance on visuo-spatial memory tests following chronic intervention with blueberries [8,9,10,11,12,13,14], strawberries [8,14], blackberries [15], grape [16], and acai berries [17]. With healthy older adults, chronic berry supplementation trials found improvements in measures of episodic memory and executive function following interventions with whole blueberry [18,19,20] and improved working memory with a mixed berry beverage containing blueberry, blackcurrant, elderberry, lingonberry, strawberry and tomato [21]. Furthermore, where the participant is suffering from cognitive decline or mild cognitive impairment, interventions have been found to be particularly effective in improving episodic memory performance following blueberry [22,23], grape [24,25], and combined grape/blueberry extract [26]. Together this evidence strongly suggests that episodic memory is particularly sensitive to flavonoid-rich berry intervention, and that these effects may be particularly strong where the participants are cognitively compromised.

In a series of studies, work from our laboratory has demonstrated the efficacy of acute blueberry intervention in children, aged between 7 and 10 years, finding positive episodic memory benefits 2 and 6 h following intervention [27,28,29] and positive executive function benefits at 2–3 h following intervention [27,29,30]. Importantly, the blueberry-related benefits were found primarily on the more cognitively demanding elements of the tasks, such as incongruent, high load Attention Network Task trials [30], or delayed recall or recognition [27,28]. Furthermore, these 2 and 6 h time points where cognitive benefits were found, correlate with known peaks in blood flow following blueberry intervention [31] indicating that the improvements found may, in part, be facilitated by increased cerebral blood flow.

To date there has been little research investigating the impact of berry interventions on cognition in young healthy adults aged between 18–30 years. Chronic goji berry intervention has been found to have a positive effect on young, 18+ years adults who reported improvements on mood related ratings of happiness, contentment, fatigue, and stress [32]. Improvements in self-reported ability to focus and mental acuity were also reported, however it should be noted that no direct cognitive behavioral tests were carried out. Two further studies have considered the cognitive effects of berry intervention following a single administration; improved composite attention function was observed 20 min after a grape juice intervention [33], and improved executive function performance was found for the rapid visual information processing task (RVIP) and digit vigilance task 70 min following acute blackcurrant intervention [34]. Furthermore, Khalid et al. [35] found increased positive affect 2 h after a blueberry intervention in young adults, aged 18–21 years. From the research available, it would therefore seem that, for young adults, benefits of blueberry intervention are primarily found within the domain of executive function with acute mood benefits also being evident.

These berry related findings are consistent with the wider flavonoid related literature. For example, in a review of acute flavonoid cognitive research [5] it was noted that whilst improvements in episodic memory might be found in children and older adults, younger adults primarily display improvements in executive function along with evidence of working memory and psychomotor processing speed. Furthermore, as discussed above, it has been observed that, where improvements are found following flavonoid intervention, they tend to be found where the individual is cognitively compromised in some way. This may be in terms of cognitive decline or MCI in older individuals [18,19,20,22,23,24,25,26], during stages of cognitive fatigue [34], or on the more cognitively demanding element of tasks, such as switching cost measures on the task switching test, or interference trials of the flanker/ modified attention network task [18,27,30].

To date, with the exception of Bensalem et al. [26] and Nilsson et al. [21], both of which were chronic studies considering aging participants, berry research on cognition and mood has focused on single source interventions. The acute effects of a combined flavonoid-rich intervention employing multiple berries on a younger adult population has yet to be considered. Furthermore, with the exception of Whyte et al. [27] with 7–10 years old children, acute interventions have only considered the effects up to 2 h following berry intervention, and no studies at all have considered the effects of berries on mood beyond 2 h. However, the berry related blood flow effects [31] would indicate that, in line with physiological effects, cognitive benefits may be found up to 6 h following intervention. The current study therefore aims to address these gaps in the literature by testing participants over 6 h following intervention using a cognitively demanding task battery. In line with the findings of Whyte et al. [30] it is hypothesized that berry-related improvements will be most evident on the more cognitively demanding trials of the tasks at all time points tested following intervention with the effects becoming particularly evident at the later stages of testing as the participants tire and cognitive fatigue increases. Given previously observed mood effects following berry intervention [35] at 2 h, similar mixed berry benefits are expected to be found here, however, given the sparsity of research relating to mood, no strong predictions are made regarding the later 4 and 6 h time points.

## 2. Materials and Methods

The study used a randomized, single blinded, placebo-controlled, parallel group design to assess the efficacy of an acute, flavonoid-rich, mixed berry intervention on executive function and mood. This study was conducted in accordance with the Declaration of Helsinki. It was reviewed by the University of Reading Research Ethics Committee and was given a favorable ethical opinion for conduct (2017-022-CW). All participants gave informed consent.

### 2.1. Participants

**Figure 1:**
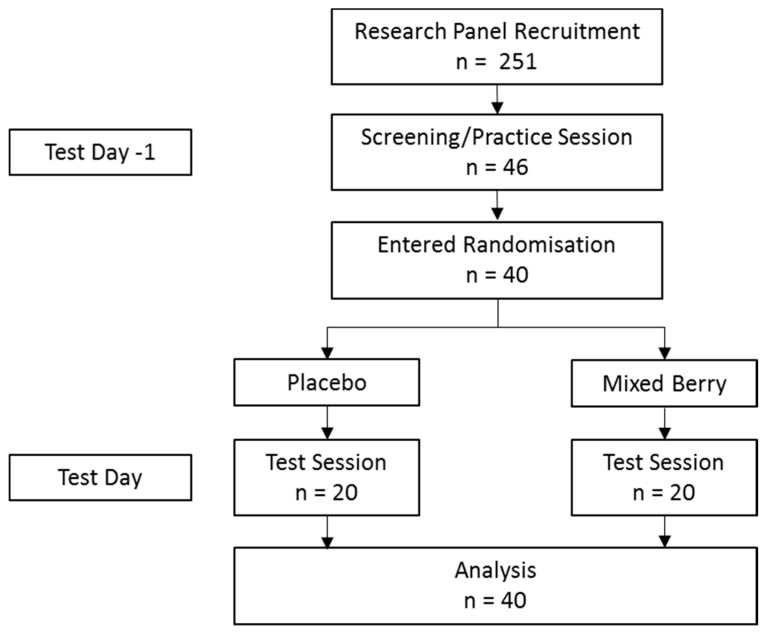
Intervention allocation and participant numbers throughout study.

Based upon a medium effect size (*d* = 0.64) observed in previous studies using grouped executive function and episodic memory domains, it was calculated that a sample size of 20 participants per experimental group would provide considerable power of 0.96. Forty-six healthy young adults were recruited to the study. Six participants withdrew from the study following Test Day-1. Therefore, forty participants completed testing and were aged between 20 and 30 years (mean = 22.8, SD = 2.6) and of all ethnicities (Figure 1). Exclusion criteria included non-native English speakers, significant vision, hearing and language problems, medical conditions such as diabetes or cardiovascular problems, and pregnancy. No significant difference was found between intervention groups on any demographic measure (Table 1).

### 2.2. Interventions

The berry intervention was a 400 mL ‘smoothie’ consisting 75 g each of whole strawberries, blueberries, blackberries, and raspberries, blended with 100 mL water and containing 14.3 g polyphenol content. The precise flavonoid content was not characterized; however, this was calculated using the USDA database, yielding the averages shown in Table 2 [7]. The two predominate flavonoids found in our intervention are anthocyanidins and proanthocyanidins along with a lower level of flavan-3-ols. The berries used in the smoothie were supplied by British Summer Fruits Association and frozen at −18 °C until point of use whereupon they were defrosted and mixed using a 500 W blender before serving. The placebo was matched to the berry intervention for carbohydrates and vitamin C and consisted of 341 mL water, 11.6 g fructose, 10.0 g glucose, and 37.4 g vitamin C. Both drinks were served in opaque flasks through opaque black straws.

### 2.3. Cognitive Tasks

The cognitive task battery lasted 30 min and consisted of the following tasks presented in the order described below:

Modified Attention Network Task (MANT) to measure selective attention under different levels of cognitive demand. Using the method described in Whyte et al. [30], participants were presented with either orienting or alerting cues followed by an array of congruent arrows—all arrows pointing in the same direction (i.e., <<<<< or >>>>>), incongruent arrows—the center arrow pointing in the opposite direction to the surrounding 4 arrows (i.e., <<><< or >><>>) or neutral—only one arrow (i.e., < or >).Trials could either be high load—an additional row of 5 arrows is presented immediately above or below giving 10 arrows in total, medium load—a single row of 5 arrows, or low load—only one arrow is presented. Additionally, trials were split into two blocks, one as a silent condition, and one as a noise condition where school playground noise was played through headphones. Participants were instructed to press the left or right arrow keys on the keyboard according to the direction of the center arrow. Incongruent, high load, noise trials are considered to be the most cognitively demanding with slower response time and decreased accuracy expected. Accuracy and response time for congruency and load were measured separately.

Task Switch Task (TST) to measure mental flexibility. This employed a modified version of the task as described in Miller et al. [18]. Participants viewed eight equally spaced radii of a circle displayed in such a way that there are four equally spaced segments above and below a bold line. A stimulus digit selected from between 1–9 (excluding 5) appeared in each segment in turn in a clockwise direction. Each digit was displayed for a duration of 3000 ms, or until the participant responded. The inter-stimulus interval was 500 ms. Dependent on whether the stimulus was above or below the bold line, participants performed different tasks. If the number was above the bold line, participants discerned whether the stimulus was odd or even by pressing the relevant response key, whereas if the number was below the bold line, participants discerned whether the number was higher or lower than 5 again by pressing the relevant key. The task therefore switches every 4 trials, with S0 denoting the initial trial following task switch and S1, S2, and S3 denoting the remaining 3 trials before the next task switch. The initial S0 trial following switch is considered to the most cognitively demanding with slower response time and increased errors expected. Accuracy and response time for task and trial following switch, were measured separately.

Positive and Negative Affect Schedule (PANAS-NOW) gives a measurement of positive and negative mood states [36,37]. Participants rate the extent to which they are experiencing 20 different emotions on a 5-point Likert scale ranging from ‘very slightly’ to ‘very much’. Half of the presented emotions relate to negative affect and half to positive affect with both scales having a maximum score of 50.

### 2.4. Procedure

The study comprised a practice/screening visit and a test visit, occurring on two consecutive days. During the practice visit, participants completed the health and wellbeing questionnaire, letter and category frequency tasks, and a complete run through of a matched practice version of the full cognitive task battery. Upon completion of screening participants were randomized to intervention.

**Figure 2:**
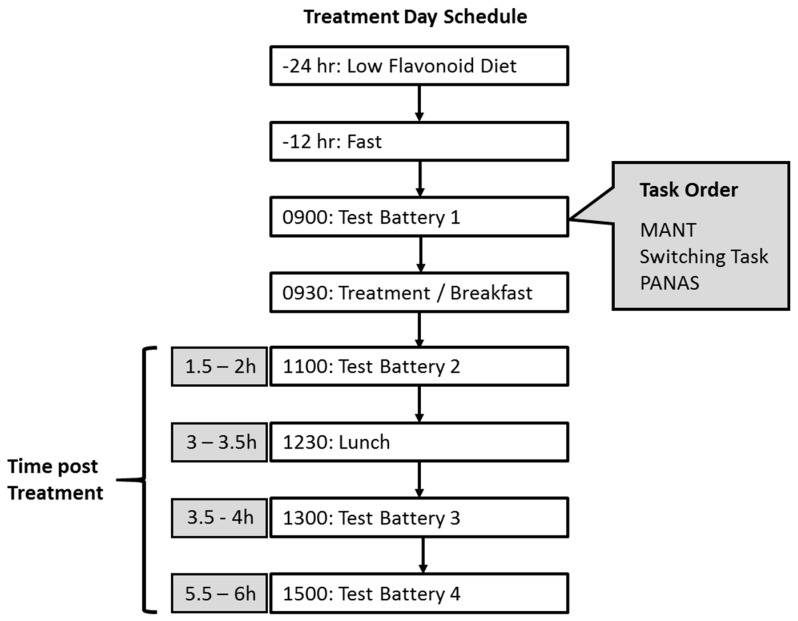
Time line of procedure and task order on intervention days.

Twenty-four h before the test visit, participants followed a dietary restriction sheet detailing high flavonoid foods to avoid consuming and refrained from vigorous physical exercise. Participants also fasted (water only) for the 12 h preceding the test visit. Upon arrival at the laboratory, participants completed a fruit and vegetable consumption questionnaire to ensure adherence to the low flavonoid diet. The cognitive task battery was completed at baseline, then 2, 4 and 6 h following intervention (see Figure 2). Immediately following baseline testing the intervention or placebo drink was consumed from an opaque flask through a black straw along with standardized low flavonoid breakfast of 2× butter croissants, 35 g light cream cheese, and water ad-libitum (391 kcal in total). A standardized low flavonoid lunch of a chicken or ham sandwich with 5 g spread, 1 × 25 g ready salted crisps, and one banana (531 kcal in total) was consumed between the 2nd and 3rd cognitive task batteries.

### 2.5. Statistical Analysis

All analysis was performed using SPSS v.22 (IBM, Chicago, USA). Linear mixed modelling was used to analyze data employing an unstructured matrix to model successive repeat measurements. Subjects were included as a random factor to control for non-independence of data within subjects. For the MANT, analyses were limited to the main effects of Intervention and Session two ways intervention × session, intervention × congruency, intervention × load, and intervention × noise interactions, and three ways intervention × session × congruency, intervention × session × load, intervention × session × noise, and intervention × congruency × load. For the Switching Task, analyses were limited to the main effects of Intervention and Session, two ways intervention × time, intervention × switch trials (S0, S1, S2, and S3), and intervention × task, and three way intervention × session × switch trials, and intervention × session × task. All analyses included baseline performance as a covariate. In all analyses, multiple pairwise comparisons with Bonferroni correction for familywise error were applied to all two and three way intervention related interactions.

## 3. Results

### 3.1. MANT Accuracy

**Figure 3:**
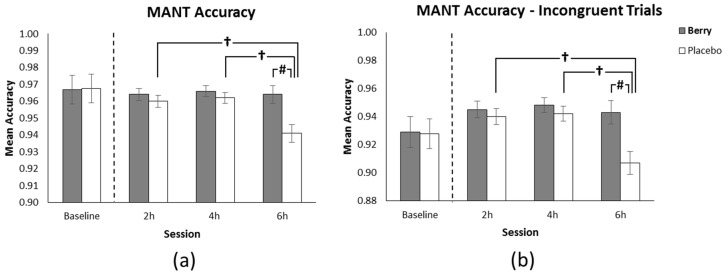
(**a**) Mean correct responses (±SE of the mean) as a function of intervention and session, showing a significant reduction in accuracy for placebo at 6 h in comparison to 2 and 4 h and significantly better performance in the berry condition in comparison to placebo at 6 h. (**b**) Mean correct incongruent trial responses (±SE of the mean) as a function of intervention and session showing a significant reduction in accuracy for placebo at 6 h in comparison to 2 and 4 h and significantly better performance in the berry condition in comparison to placebo at 6 h. Baseline performance, included as a covariate in the analysis, is shown on both graphs separated by the dotted line. # *p* < 0.01, † *p* < 0.001.

The omnibus analysis revealed a significant main effect of intervention (F(1396) = 2.92, *p* = 0.024) with participants in the berry condition performing more accurately than placebo. This main effect was qualified by an intervention x session interaction trend (F(2400) = 2.472, *p* = 0.086) along with a trend towards significance for session (F(2400) = 2.92, *p* = 0.055). Post hoc analysis of the intervention × session interaction revealed more accurate performance in the berry condition in comparison to placebo at 6 h following intervention (*p* = 0.003). Furthermore, accuracy following placebo reduced significantly between 2 h (*M* = 0.96) and 6 h, and between 4 h and 6 h (both *p* < 0.001) whereas performance in the berry condition remained constant across the day (see Figure 3a).

A significant intervention x congruency interaction was also revealed (F(2405) = 27.6, *p* < 0.001); post hoc analysis found no difference between interventions for the congruent and neutral trials, however on the more cognitively demanding incongruent trials, participants in the berry condition performed more accurately than placebo. Additional post-hoc analysis further qualified this finding by revealing no difference between interventions on the incongruent trials at 2 and 4 h following intervention, however, berry performance was significantly more accurate on incongruent trials at 6 h in comparison to placebo (*p* = 0.002). Furthermore, placebo intervention accuracy on incongruent trials reduced significantly between 2 h and 6 h, and 4 h and 6 h (both *p* < 0.001) whereas performance in the berry condition remained constant across the day (see Figure 3b). This finding indicates the effects of the intervention x session interaction described above were primarily driven by poorer placebo performance on the more cognitively demanding incongruent trials.

### 3.2. MANT Response Time

**Figure 4:**
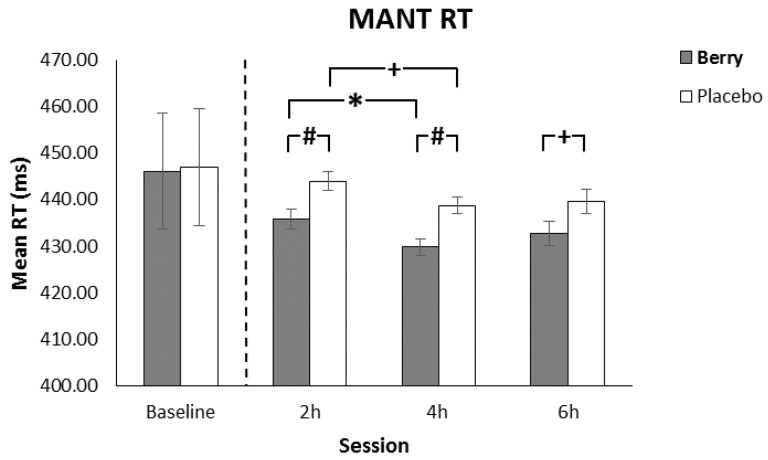
Reaction time (± SE of the mean) as a function of intervention and session, showing significantly faster berry performance at 2 and 4 h in comparison to the placebo and a significantly faster berry performance between 2 and 4 h. Note there was a trend towards significance at 6 h for faster berry performance in comparison to the placebo and a trend for faster placebo performance between 2 and 4 h. Baseline performance, included as a covariate in the analysis, is shown separated by the dotted line. + *p* < 0.1, * *p* < 0.05, # *p* < 0.01.

The omnibus analysis revealed a significant main effect of intervention (F(1397) = 5.96, *p* < 0.001) with participants in the berry condition responding faster than placebo along with a main effect of session (F(2400) = 5.96, *p* = 0.003). As can be seen in Figure 4, post hoc analysis revealed significantly faster performance following the berry intervention in comparison to placebo at 2 h (*p* = 0.006) and 4 h (*p* = 0.001) with a further trend for faster berry performance at 6 h (*p* = 0.068); furthermore significantly faster response times were seen at 4 h in comparison to 2 h for the Berry intervention (*p* = 0.021) whilst the placebo trended towards significance for the same time period (*p* = 0.051).

The main effect of intervention was qualified by intervention × congruency (F(2408) = 48.0, *p* < 0.001] and intervention × load (F(2398) = 3.23, *p* = 0.041) interactions. For the intervention × congruency interaction, post-hoc analysis revealed faster response times for the berry intervention in comparison to placebo for incongruent trials (*p* = 0.047) and neutral trials (*p* = 0.022). Specifically, on neutral trials significantly faster response times for the berry intervention, in comparison to placebo, were seen at 2 h (*p* = 0.031) and 4 h (*p* = 0.030), whilst for incongruent trials significantly faster berry-induced responses were seen at 4 h only (*p* = 0.007). There was also trend for faster berry response times in comparison to the placebo on congruent trials seen at 2 h (*p* = 0.087). Furthermore, there were significantly slower response times for the placebo condition on congruent trials at 4 h in comparison to 2 h (*p* = 0.021) whilst speed of berry performance was maintained between these sessions.

For the intervention × load interaction, post-hoc analysis revealed significantly faster berry performance, in comparison to placebo, for medium (*p* = 0.026) and low (*p* = 0.022) load trials. Additional post-hoc analysis further qualified this finding by revealing significantly faster berry response times in comparison to placebo on low load trials at 2 h (*p* = 0.031) and 4 h (ms; *p* = 0.030), and also medium load trials at 4 h (*p* = 0.002). Furthermore, there were significantly faster response times at 4 h in comparison to 2 h on medium load trials for the Berry intervention (*p* = 0.031) whilst there was no such benefit for the placebo. The results here demonstrate better response time performance for the berry intervention in general with benefits particularly evident at the 2 and 4 h sessions. The predicted cognitive demand/fatigue effects were not found for these response time measures with the benefits being shown on a spread of congruent, incongruent, low load, and medium load trials. This indicates that the response times effects found here are more global in nature in contrast to the accuracy benefits which were found on the more cognitively demanding trials as outlined above.

### 3.3. Switching Task Accuracy

**Figure 5:**
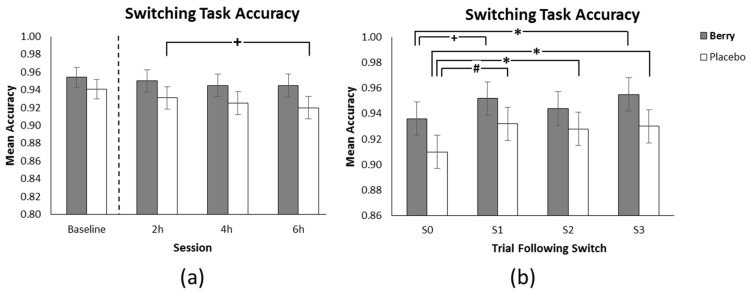
(**a**) Mean correct responses (±SE of the mean) as a function of intervention and session. (**b**) Mean correct responses (±SE of the mean) as a function of intervention and switch trial showing a significant switch cost between S0 and S1, S2, and S3 for placebo and a significant switch cost only between S0 and S3 for berry. Baseline performance, included as a covariate in the analysis, is shown on graph A separated by the dotted line. + *p* < 0.1, * *p* < 0.05, # *p* < 0.01.

The omnibus analysis revealed no significant main effect for intervention type, however, there was a significant intervention x switch trial interaction (F(6270) = 4.1, *p* = 0.001), a trend towards significance for the intervention x task x session interaction (F(4320) = 2.18, *p* = 0.070) and a main effect of session (F(2320) = 3.63, *p* = 0.028). As shown in Figure 5a post hoc analysis revealed a trend towards significance whereby there was a reduction in accuracy for placebo between 2 h and 6 h (*p* = 0.050).

As shown in Figure 5b post hoc analysis of the intervention x switch trial interaction revealed that for placebo there was a significant difference between S0 and S1 (*p* = 0.004), S2 (*p* = 0.027) and S3 (*p* = 0.012), however following the berry intervention there was only a significant difference between S0 and S3 (*p* = 0.016), and a weak trend between S0 and S1 (*p* = 0.084) indicating less evidence of a cognitive demand related switching cost (i.e., the difference between the S0 trial immediately following task switch and the following S1, S2, and S3 non-switch trials).

Post-hoc analysis of the intervention × task × session interaction revealed no significant pairwise comparisons, however, for placebo, there was a trend for less accurate performance at 6 h in comparison to 4 h on the high/low task (*p* = 0.056) along with a weak trend for less accurate performance at 4 h in comparison to 2 h on the odd/even task (*p* = 0.092). For the berry treatment there was a weak trend for less accurate performance at 6 h in comparison to 2 h on the odd/even task (*p* = 0.094). A further trend was found for less accurate placebo performance in comparison to the berry treatment on the high/low task at 6 h (*p* = 0.078).

### 3.4. Switching Task Reaction Time

**Figure 6:**
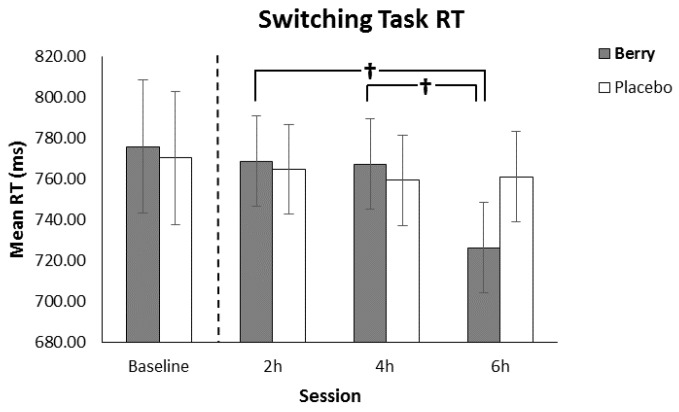
Reaction time (±SE of the mean) as a function of intervention and session, showing significantly faster performance at 6 h in comparison to 2 and 4 h for the berry intervention. Baseline performance, included as a covariate in the analysis, is shown separated by the dotted line. † *p* < 0.001.

The omnibus analysis revealed no significant main effect for intervention, however, there was a significant intervention × session interaction (F(2320) = 6.73, *p* = 0.001), a significant intervention × switch trial interaction (F(6275) = 9.60, *p* < 0.001), a trend towards an intervention × task interaction (F(2264) = 2.44, *p* = 0.089), and main effect of session (F(2320) = 7.37, *p* = 0.001). Post hoc analysis of the intervention × session interaction revealed a significant decrease in response time for the berry intervention between 2 h and 6 h (*p* < 0.001) and 4 h and 6 h *p* < 0.001) indicating improved reaction times at the point participants would be expected to be most cognitively fatigued (see Figure 6). No such benefit was found for the placebo. Post hoc analysis of the intervention x switch trial interaction found significant reductions between switch trial 0 and trials 1, 2, and 3 for both interventions (all *p* < 0.001). Post hoc analysis of the intervention × task interaction revealed a significant difference between the high/low and odd/even tasks for the berry intervention (*p* = 0.029) whereas no such difference was found for the placebo.

### 3.5. Positive Affect

**Figure 7:**
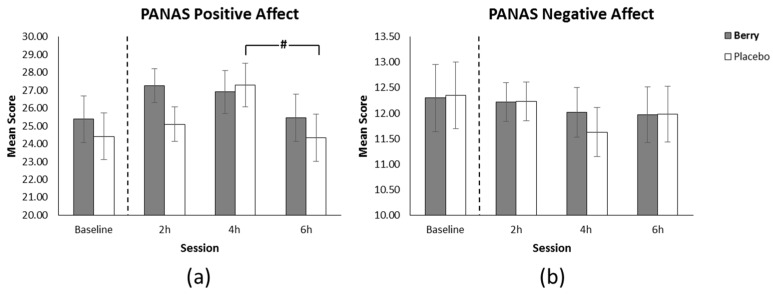
(**a**) Positive affect scores (±SE of the mean) as a function of intervention and session, showing a significant reduction at 6 h in comparison to 4 h for the placebo intervention. (**b**) Positive affect scores (±SE of the mean) as a function of intervention and session showing no difference between interventions at any time points.

The omnibus analysis revealed a significant main effect whereby session predicted positive affect (F(140) = 4.71, *p* = 0.015). There was no significant intervention related main effect or interaction. (see Figure 7a).

### 3.6. Negative Affect

The omnibus analysis revealed no significant main effects, interactions, or pairwise comparisons. (see Figure 7b).

## 4. Discussion

This study considered the effects of a flavonoid rich berry intervention on cognition and mood in young 20–30 years old adults. It was hypothesized that cognitive benefits of berry intervention would be found on the more demanding trials of the executive function tasks or where participants were cognitively fatigued. Berry intervention was also expected to facilitate improved positive affect at 2 h.

As predicted, following placebo intervention, there was a significant decrease in accuracy at the 6 h time point for the MANT and a trend for the same pattern on the Switching Task. In both cases this was overcome by our berry intervention where there was no decrease in accuracy performance. This effect was particularly evident on the MANT where at 6 h, berry accuracy was significantly better than placebo. Similarly, it was predicted that there would be an increase in response time following placebo intervention. Whilst this effect was not found, participants in the berry condition showed significantly faster response time at the 6 h time point whilst performing the Switching Task. The 6 h test session took place at 15:00 h during the known post lunch dip in cognitive performance and also nearing the end of the working day when participants would be most cognitively fatigued. It is therefore particularly interesting that the berry intervention not only maintained accuracy at this point but facilitated an improvement in response times. Importantly, there is a known peak in blood flow at 6 h following blueberry intervention [31] and this may have ameliorated the detrimental effects of cognitive fatigue and facilitated improved performance following berry intervention. Furthermore, the oligomeric proanthocyanidin content of the berries is also high, as shown in Table 2. Whilst monomeric flavan-3-ols can be absorbed through the small intestine and found in plasma as early as 1.5 h following ingestion [38], the oligomeric proanthocyanins are broken down in the colon starting at ~6 h. It has been proposed that the resulting phenolic acids are responsible for further biological effects [39] and may therefore have contributed to the improved cognitive function found at 6 h in the present study. Likewise, other polyphenol content of the berries, not characterized here, may be metabolized and have an effect at different time points throughout the post intervention period.

As was predicted there was evidence that the beneficial effects found at 6 h on the MANT were primarily driven by better berry performance on the more cognitively demanding incongruent trials of this executive function task. Furthermore, following berry intervention, there was evidence of reduced switching cost between the initial S0 and S1/S2 trials across all time points, an effect which was not seen for the placebo. This finding gives further support to our previous findings indicating flavonoid-rich interventions are most effective where cognitive demand is high [30]. However, it should be noted that berry-related accuracy benefits were not found for the MANT at the 2 h time point where peaks in blood flow are also known to occur. Between 20–30 years, brain development concludes and cognitive ability peaks.

In the current study, accuracy for the MANT approached ceiling with participants performing at 96% accuracy at 2 h. In contrast, 7–10 years old children performing on this task typically perform at 79–85% accuracy [29]. Whilst still fresh at the early 2 h stage of testing, our healthy young adult sample in both treatments were performing near ceiling leaving little room for benefits of the intervention to manifest themselves. Accuracy for the placebo intervention, however, dropped to 94% at 6 h indicating the combination of fatigue and cognitive demand had a greater detrimental effect which the berry intervention was able to overcome. Whilst this finding indicates an overall difference of 2% between the placebo and berry conditions, as shown in Figure 4 above, this difference increases to 3.5% when comparing the more cognitively demanding, incongruent trials. In terms of practical significance, though these benefits are small in absolute terms, when considered alongside the maintenance or improvements in reaction time performance found for the berry treatment, it can be argued that these results are promising in terms of maintenance of performance which might be particularly beneficial in occupations which require sustained attention over extended durations under distracting conditions.

For the MANT, response times improved from 2 to 4 h. Whilst this was significant following berry intervention, the trend for improved placebo response times over the same period raises the possibility that the faster performance for both interventions may have been a result of practice effects. Importantly, there was no significant change in response times between 4 and 6 h indicating that there was no speed/accuracy trade off underlying the improved accuracy performance reported above at 6 h. Furthermore, when the interventions were compared directly, significantly faster berry response times were found in comparison to placebo at 2 and 4 h along with a trend for faster berry performance at 6 h demonstrating a berry intervention benefit over and above any practice related improvements. This is in keeping with previous flavonoid rich intervention studies, where response time benefits were found 2 h following blueberry treatment with 7–10 years old children on the MANT [29,30] and shows that the cognitive benefits indicated by improved performance on this task can be extended to a young adult population, even in the absence of cognitive fatigue.

Contrary to our hypotheses, though positive affect was higher for blueberry than placebo at 2 h following intervention, this finding failed to reach significance. Whilst previous research has shown improvements in positive affect in young adults (aged 18–25) 2 h following blueberry intervention [35] it should be noted that these benefits were found following a dose equivalent to over 200 g of fresh blueberry, whereas a different profile of multiple berries were used in the current study. It is therefore possible that the particular flavonoid profile of the mixed berry intervention was not sufficient to engender a change in mood.

In terms of the intervention itself, though there is a growing body of research demonstrating the positive cognitive benefits of berries in general, no direct claims can be made regarding the specific effects of the individual berries contained in the intervention used here. Further research with separate and combined arms is required to elucidate the individual effects of each berry type and also indicate whether there may be an additive or synergistic effect of the berries in combination. Proposed mechanisms through which flavonoids exert beneficial effects on measures of cognitive performance include improved cerebrovascular blood flow and mediation of cell signaling pathways [40]. The flavonoid profiles of each individual berry should therefore be taken into account alongside measures of physiological biomarkers with a view to correlating metabolic and cognitive changes. Furthermore, the current study considered the effects of the berry intervention acutely on only one age group therefore, at present, no direct statements can be made regarding efficacy in other age groups. However, given that the benefits became evident when the participants were cognitively compromised, it is feasible that a similar effect may be found in older adults subject to cognitive decline. At 300 g, the quantity of berries consumed in this study, is equivalent to 1¾ portions and is perhaps more than one would normally consume at a single meal. An acute dose response study would therefore be recommended in order to ascertain whether benefits might be found at more typical levels of consumption. Though previous chronic research with older adults has demonstrated working/episodic memory following mixed-berry interventions [21,26], this was outside the scope of the current study. A further chronic study considering multiple cognitive domain outcomes with our mixed berry treatment is therefore recommended to investigate the extent of cognitive benefits in younger adults, alongside consideration of tolerance effects and whether benefits extend beyond the point intake is discontinued.

Under the constraints of the current study, preparation and administration of the drinks was performed by a single experimenter. It was therefore not possible to double-blind this study by dividing these processes between separate personnel. In order to keep participants blind to which intervention was received, the drinks were served in opaque flasks with opaque black straws. The placebo was matched to the berry intervention for sugar content and sweetness, but not berry flavor. It is therefore possible that some participants may have guessed which treatment they received leading to performance being influenced by a placebo effect. A replication study utilizing a more closely taste and texture matched placebo is recommended.

## 5. Conclusions

This study adds further support to the growing evidence that acute intervention with flavonoid rich berries facilities improvements in positive affect and cognitive executive function. The cognitive effects are particularly evident during periods of cognitive compromise where the individual is fatigued and task demand is high.

## Figures and Tables

**Table 1:** Demographic details by intervention group standard deviation in parenthesis.

	Age	Alcohol Units/Week	Tea/Day	Exercise Hours/Week	Letter Fluency	Category Fluency
Placebo	22.8 (2.8)	3.24 (3.44)	0.88 (0.96)	2.73 (2.26)	16.55 (3.52)	22.1 (4.35)
Berry	22.8 (2.46)	5.07 (5.74)	1.25 (1.47)	4.30 (3.24)	15.25 (4.44)	19.65 (4.74)
*p*-value	1.00	0.229	0.347	0.083	0.311	0.097

**Table 2:** Average flavonoid content mg/75 g by flavonoid class and berry type [7].

	Antho-Cyanidins	Flavan-3-ol	Flavanone	Flavone	Flavonol	Proantho-Cyanidin	Total
Raspberry	36.45	4.35	0	0	0.9	26.9	68.6
Blueberry	122.4	5	0	0.15	8	136.5	272.05
Blackberry	75.5	32	0	0	3.45	14	124.95
Strawberry	20.3	3.37	0.23	0	1.2	79	104.1
Total	254.65	44.72	0.23	0.15	13.55	256.4	569.7
